# Muscle fatigue, pedalling technique and the ${\dot{V}}_{O_{2}}$ slow component during cycling

**DOI:** 10.1113/EP092116

**Published:** 2024-10-16

**Authors:** Keenan B. MacDougall, Saied J. Aboodarda, Paulina H. Westergard, Brian R. MacIntosh

**Affiliations:** ^1^ Faculty of Kinesiology University of Calgary Calgary Canada

**Keywords:** cycling efficiency, electromyography, energetics, oxygen uptake, ${\dot{V}}_{O_{2}}$ kinetics

## Abstract

Above the first lactate threshold, the steady‐state ${\dot{V}}_{O_{2}}$ is delayed or prevented due to the ${\dot{V}}_{O_{2}}$ slow component (${\dot{V}}_{O_{2}\mathrm{SC}}$). This phenomenon has been associated with muscle fatigue, but evidence for a causal relationship is equivocal. Moreover, little is known about the contribution of pedalling technique adjustments to ${\dot{V}}_{O_{2}\mathrm{SC}}$ during fatiguing cycling exercise. Eleven participants completed constant power trials at 10% above the second lactate threshold. Muscle fatigue was assessed, utilizing femoral nerve stimulation and instrumented pedals, while ${\dot{V}}_{O_{2}}$, quadriceps oxygenation, electromyography (EMG) and pedal force components were measured. Correlations between physiological and mechanical variables were estimated at group and individual levels. Group correlations revealed moderate values for ${\dot{V}}_{O_{2}\mathrm{SC}}$ with quadriceps twitch force (*r *= −0.51) and muscle oxygenation (*r *= −0.52), while weak correlations were observed for EMG amplitude (*r *= 0.26) and EMG mean power frequency (*r *= −0.16), and with pedalling mechanical variables such as peak total downstroke force (*r *= −0.16), minimum total upstroke force (*r *= −0.16) and upstroke index of effectiveness (*r *= 0.16). The findings here align with prior literature reporting significant correlations between the magnitude of muscle fatigue and that of ${\dot{V}}_{O_{2}\mathrm{SC}}$, although there was large interindividual variability for all the reported correlations. Considering the heterogeneity in the data, it is difficult to determine the relative impact of pedalling technique adjustments on ${\dot{V}}_{O_{2}\mathrm{SC}}$ overall, but the present study opens the possibility that in some cases, increases in ${\dot{V}}_{O_{2}}$ secondary to technical adjustments may be ‘superimposed’ on the underlying ${\dot{V}}_{O_{2}\mathrm{SC}}$.

## Abstract (highlights)

- **What is the central question of this study?**
  Is there an interplay between muscle fatigue and alterations in pedalling technique in the development of the ${\dot{V}}_{O_{2}}$ slow component during cycling?
- **What is the main finding and its importance?**
  There was a significant correlation between the ${\dot{V}}_{O_{2}}$ slow component and muscle fatigue, although they were not temporally aligned, and substantial individual heterogeneity existed in the strength of the association. Alterations in pedalling technique were shown to be weakly correlated with the ${\dot{V}}_{O_{2}}$ slow component across the whole group, but certain individuals showcased strong associations, suggesting that biomechanical changes may play a role in the development of the ${\dot{V}}_{O_{2}}$ slow component in some individuals.

## 1 INTRODUCTION

It has long been known that when exercising at intensities above the first lactate threshold, the steady‐state ${\dot{V}}_{O_{2}}$ is delayed compared to what is observed at lower intensities, or may not be reached at all. Whipp and Wasserman (1972) were amongst the first to categorically separate the ${\dot{V}}_{O_{2}}$ response into a rapid and a slower component, in which the slower component only became evident after approximately 3 min of exercise. Today, this is commonly known as the slow component of oxygen uptake, and, despite an enormous amount of research, there is still much disagreement on its underlying mechanism(s). The ${\dot{V}}_{O_{2}}$ slow component (${\dot{V}}_{O_{2}\mathrm{SC}}$) has been attributed to ‘a time‐dependent, as well as intensity‐dependent, loss of muscle efficiency’, assumed to be underpinned by skeletal muscle fatigue (Jones et al., 2011). In support of this notion, both Keir et al. (2016) and de Almeida Azevedo et al. (2022) reported significant temporal correlations between measures of muscle fatigue and the magnitude of the slow component, arguing for some common physiological underpinning between the two processes. Contrary to these observations, however, the progression of fatigue and the slow component have also been shown to be temporally misaligned (Cannon et al., 2011; do Nascimento Salvador et al., 2023; Gajanand et al., 2020), challenging whether the association between these processes is actually a causal one. For example, Cannon et al. (2011) demonstrated that following 3 or 8 min of heavy and very heavy constant power output cycling, peak power output significantly decreased *prior* to the development of the slow component, yet did not continue to decrease, despite an increase in ${\dot{V}}_{O_{2}}$. These investigators thus concluded that muscle fatigue must be a prerequisite for the slow component. On the other hand, and in spite of reporting significant correlations between the two measures, Gajanand et al. (2020) reported that the onset of the slow component preceded the development of any muscle fatigue, and concluded that fatigue is evidently not required to elicit the slow component. More recently, using a similar experimental set‐up to that of Cannon et al. (2011), do Nascimento Salvador et al. (2023) reported no association between muscle fatigue and the slow component.

One explanation for the discrepant findings could be key methodological differences in the assessment of fatigue. For example, some studies used performance measures such as reductions in cycling peak power or torque (Cannon et al., 2011; do Nascimento Salvador et al., 2018, 2023) while others relied on electrically evoked muscle forces (de Almeida Azevedo et al., 2022; Gajanand et al., 2020; Keir et al., 2016). Additionally, differences in the magnitude of muscle potentiation in the electrically evoked forces (Kufel et al., 2002; Keir et al., 2016; Gajanand et al., 2020), as well as the duration of the delay in the post‐fatigue assessments could impact the measured level of fatigue and lead to disparate results. In any case, despite the commonly held notion that the slow component is underpinned by muscle fatigue, the literature in support of this is far from conclusive. In the present study, we utilize a novel method of assessing quadriceps muscle fatigue by using femoral nerve stimulation during the pedalling action (MacDougall et al., 2024). This method affords a greater temporal resolution of muscle fatigue measurements compared to prior studies, improving our ability to observe the association between muscle fatigue and the slow component.

An overlooked aspect of fatigue that may also contribute to the slow component is alterations in movement strategy during the fatiguing task (Jones, 2023). With respect to cycling exercise, it is conceivable that alterations in factors such as the direction of force application on the pedals, or the relative contributions of force from the legs working on the downwards vs. upwards portion of the pedal stroke may change across an exercise bout, potentially affecting the energy cost of exercise, despite the maintenance of a constant mean power output. Indeed, both Sanderson and Black (2003) and Dorel et al. (2009) reported significant increases in peak pedal forces from the beginning to the end of a fatiguing constant power output cycling trial. While neither of these studies assessed the energetic cost associated with these alterations in technique, other studies have shown that acute alterations in pedalling technique can significantly impact the energy cost of cycling exercise (Cannon et al., 2007; Korff et al., 2007). Despite these considerations, however, the link between alterations in pedalling technique and ${\dot{V}}_{O_{2}\mathrm{SC}}$ has never been evaluated.

Therefore, the purpose of this study was to further explore the association between ${\dot{V}}_{O_{2}\mathrm{SC}}$ and skeletal muscle fatigue, as well as any association between the slow component and alterations in pedalling technique. It was hypothesized that the development of ${\dot{V}}_{O_{2}\mathrm{SC}}$ would be associated with both muscle fatigue and alterations in pedalling technique.

## 2 METHODS

### 2.1 Participants

Eleven recreationally active participants (*n* = 8 males, *n* = 3 females, mean (standard deviation): age 25 (6) years, height 174 (9) cm, weight 72 (11) kg, ${\dot{V}}_{O_{2}\mathrm{peak}}$ = 43.1 (9.8) mL kg^−1^ min^−1^) participated in this study. All participants cleared the physical activity readiness questionnaire (PAR‐Q+) and were free of any health conditions or injuries that could prevent them from maximally exerting themselves. All participants provided written informed consent before commencing the study, which was approved by the University of Calgary Conjoint Health Research Ethics Board (REB21‐1749). The study was conducted in accordance with the *Declaration of Helsinki* (without registration).

### 2.2 Experimental protocol

Participants reported to the laboratory for four visits, separated by at least 48 h. Testing was performed at a similar time of day across sessions (range 0–3 h), and participants were instructed to refrain from strenuous physical activity 24 h prior to testing, and to avoid caffeine 12 h prior to testing. The first visit included step incremental test to exhaustion to determine the lactate threshold and ${\dot{V}}_{O_{2}\mathrm{peak}}$, and served to familiarize participants with the electrical stimulation procedures. The following three visits required cycling to task failure at a constant power output 10% above the estimated power output at the second lactate threshold. Task failure was defined as when participants were not able to maintain a cadence greater than 75 rpm for 5 s despite strong verbal encouragement, or volitional exhaustion.

### 2.3 Exercise testing

For the step incremental test, participants began cycling at a power output of 50 W, and power output was increased 25 W every 3 min until volitional exhaustion. Blood lactate concentration was measured via fingertip blood samples taken at the end of each stage (Lactate Plus, Nova Biomedical, Waltham, MA, USA) and plotted against power output. The second lactate threshold was estimated using the modified Dmax method (Bishop et al., 1998), in which the power output preceding a rise in blood lactate concentration ≥0.4 mmol L^−1^, and all subsequent values were fit with a third‐order polynomial curve. The second lactate threshold was identified as the point on the curve that yielded the maximum perpendicular distance from a straight line connecting the first and final data points. This method of identifying the second lactate threshold was chosen over other methods because, as part of a separate project, we required that some of our data be measured across a variety of discrete power outputs, which a ramp incremental test would not have allowed. As a point of reference, Jamnick et al. (2018) found that the power output associated with this particular modified DMax method was, on average, ∼5% higher than that associated with the maximal lactate steady state.

The three constant power output trials were preceded by a 3‐min warm‐up at 50 W, with a step increase to the prescribed power output. The mean (standard deviation) power output for these trials was 194 (55) W, which represented 80.1 (6.0)% of the highest power output achieved during the incremental test (range 70.3–89.2%). On two of the testing occasions, skeletal muscle fatigue was assessed by a novel method in which femoral nerve stimulation was applied while the participant continued to pedal. This method allows measurement of muscle fatigue while the slow component develops, and avoids recovery of muscle force when force measurement is delayed. In addition, electromyography (EMG), near‐infrared spectroscopy (NIRS), and oxygen uptake measurements were made (see ‘Measurements and data analysis’ below). These duplicate trials were performed to assess the test–retest reliability of this novel method of fatigue assessment. At 5‐min intervals throughout the trial, participants stopped pedalling for 15–30 s and a block was placed under the right pedal to obtain resting isometric twitch measurements. These isometric twitch force data are not reported here. A third constant power output trial was also performed in which only oxygen uptake was measured (i.e., femoral nerve stimulation was not applied, and pedal forces were not measured). The ${\dot{V}}_{O_{2}}$ data from the first 5 min of this trial were used to improve the signal‐to‐noise ratio for estimating the primary phase amplitude and onset of ${\dot{V}}_{O_{2}\mathrm{SC}}$ (Jones & Poole, 2013) (see ‘Oxygen uptake’ below). Participants were instructed to maintain a strict cadence of 80 rpm throughout each trial. All testing was performed on an electromagnetically braked cycle ergometer (Velotron RacerMate, SRAM LLC, Chicago, IL, USA).

### 2.4 Measurements and data analysis

#### 2.4.1 Oxygen uptake

${\dot{V}}_{O_{2}}$ was measured breath‐by‐breath with a metabolic cart (Quark CPET, Cosmed, Rome, Italy). Breath‐by‐breath ${\dot{V}}_{O_{2}}$ data for each trial were first cleaned by removing the data points during and 60 s following the intermittent breaks required for isometric twitch measurements. Next, breaths lying outside of the 95% prediction limits of a preliminary fitting of the data were removed, and then data were linearly interpolated to 1 s intervals (Maturana et al., 2021). From the incremental test, ${\dot{V}}_{O_{2}\mathrm{peak}}$ was determined as the highest 20 s rolling average across the trial. For the modelling of ${\dot{V}}_{O_{2}}$ kinetics, the data for each of the three constant power output trials were ensemble‐averaged to provide a single dataset for each participant, time aligned such that time 0 marked the onset of the transition, and averaged into 5 s bins (Cannon et al., 2011). The first 20 s of data were removed, and the data up to 300 s were then fit to the following equation:

$${\dot{V}}_{O_{2}(t)}={\dot{V}}_{O_{2}\mathrm{BL}}+A_{p}\left[1-e^{-\left(\frac{t-\mathrm{TD}}{\tau }\right)}\right]$$

where ${\dot{V}}_{O_{2}(t)}$ is the ${\dot{V}}_{O_{2}}$ at any time point, ${\dot{V}}_{O_{2}\mathrm{BL}}$ is the baseline ${\dot{V}}_{O_{2}}$, calculated as the mean ${\dot{V}}_{O_{2}}$ for the 60 s leading up to the step transition, *A*_p_ is the amplitude of the primary component, TD is the time delay of the response, τ is the time constant of the response, and *e* is the base of the natural logarithm function (Barstow & Mole, 1991). An iterative process was used for determining the fitting window to isolate the primary (phase II) ${\dot{V}}_{O_{2}}$ response and the subsequent onset of ${\dot{V}}_{O_{2}\mathrm{SC}}$, in which the end of the fitting window was increased from 80 s up until 300 s, and the estimated *A*_p_, τ, TD and χ^2^ values for the fitted model were plotted against the fitting window duration. The end of the primary phase and the onset of the slow component were determined by identifying coincident upward breakpoints in the plots for *A*_p_, τ and χ^2^, and a downward breakpoint for TD (Murgatroyd et al., 2011). During the constant power output trials, 20 s bin averages for ${\dot{V}}_{O_{2}}$ were taken at the mid‐ and end‐points of each 5 min period (between 2:10 and 2:30 and 4:40 and 5:00 of each period, i.e., approximately every 2.5 min, not including the brief 15–30 s periods when isometric twitch measurements were being made), and just prior to task failure. At each time point, the magnitude of ${\dot{V}}_{O_{2}\mathrm{SC}}$ was measured as the difference between the 20 s mean ${\dot{V}}_{O_{2}}$ and the projected primary component steady state (i.e., the sum of the ${\dot{V}}_{O_{2}\mathrm{BL}}$ and *A*_p_) (Cannon et al., 2007; de Almeida Azevedo et al., 2022; Keir et al., 2016). During time points prior to the estimated onset of ${\dot{V}}_{O_{2}\mathrm{SC}}$ (e.g., at exercise onset and sometimes between the 2:10 and 2:30 min time point), or if the measured ${\dot{V}}_{O_{2}}$ was less than the projected primary component steady state, the ${\dot{V}}_{O_{2}\mathrm{SC}}$ was given a value of zero.

#### 2.4.2 Muscle fatigue

**FIGURE 1:**
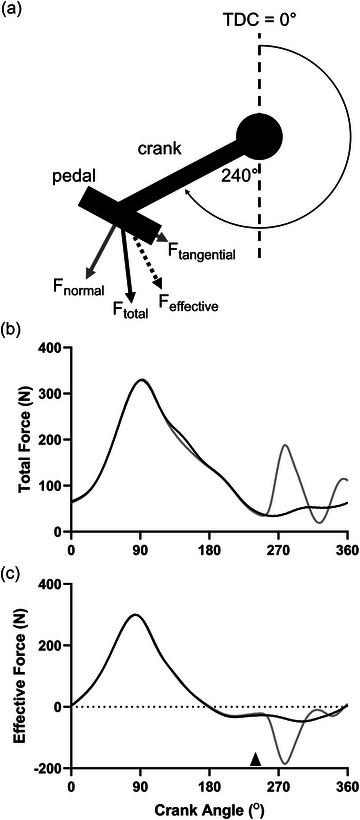
Pedal force derivations and representative force profiles across a full pedal revolution. (a) The various force components applied to the pedal. The total applied force (*F*_total_) is calculated as the vector sum of the normal (*F*_normal_) and tangential (*F*_tangential_) forces acting on the pedal, and the effective force (*F*_effective_) is the component of *F*_total_ acting perpendicular to the crank. (b, c) Representative force traces across a full pedal revolution from one participant for *F*_total_ and *F*_effective_. The grey traces show the mean force profile from three revolutions where twitches were evoked to illustrate how muscle fatigue was measured across the trials, while the black traces show the mean force profile from 10 revolutions prior to the stimulations. Stimulation occurred at a crank angle of 240° (denoted by the black arrow in (c)).

To measure forces applied to the pedals, the ergometer was outfitted with triaxial instrumented pedals (Sensix, Poiters, France), which were sampled at 500 Hz per channel. To measure skeletal muscle fatigue, single square‐wave electrical stimuli (pulse duration: 1 ms; 400 V) were delivered to the right femoral nerve percutaneously using a constant‐current stimulator (DS7A; Digitimer, Welwyn Garden City, UK). The cathode (10 mm diameter; Kendall MediTrace; Covidien LLC, Mansfield, MA, USA) was placed on the femoral triangle, and the anode (50 × 90 mm; Durastick Plus; DJO Global, Vista, CA, USA) was placed approximately halfway between the greater trochanter and the iliac crest. At the beginning of each session, with participants seated on the bike and a block placed underneath the right pedal at a crank angle of 90° (0° = top dead centre), the current intensity was increased in 10–20 mA increments until a plateau in twitch force was observed. The current intensity was then increased by 30% to ensure complete motor unit activation during stimulation, and this intensity was used for the remainder of the session. The mean ± standard deviation stimulation current used for all sessions was 109 ± 21 mA. To measure quadriceps twitch force during cycling, the cycle ergometer was outfitted with a custom‐made photoelectric switch mounted on the seat tube. On the inside of the chain ring, a piece of reflective paper triggered the switch during the upstroke portion of the pedal cycle at a crank angle of 240° (0° = top dead centre). The switch was connected to the electrical stimulator, as well as a PowerLab data acquisition device (Model 8/35, ADInstruments Inc., Colorado Springs, CO, USA) in order to set the minimum time interval between successive stimulations. This technique results in a brief ‘counterforce’ action during the upstroke portion of the pedal cycle, but any disruptions to the pedalling rhythm were negligible. For all twitch force measurements, the total force applied to the pedals (*F*_total_) was used for analysis (see ‘Pedalling mechanical variables’ and Figure 1). Twitch amplitudes were calculated by subtracting a baseline force estimated from linearly interpolating the force between the onset and offset of the twitch, identified as the points at which the slope of the force trace was equal to zero, and solving for the time at which the peak twitch force occurred (MacDougall et al., 2024). Twitch force measurements were obtained at the very beginning of the bout, as soon as participants reached the proper cadence (between ∼10 and 20 s into the bout), at the mid‐ and end‐points of each 5‐min period (between ∼2:15 and 2:30 and ∼4:45 and 5:00 of each period, respectively), as well as prior to task failure, following the participant signalling they were nearing completion. At each time point, three separate stimuli were given ∼3 s apart, and the mean of these measurements was used. The reliability of this method was shown to be commensurate with twitch measurements made on traditional seated isometric dynamometers (MacDougall et al., 2024). In two instances, the stimulating electrode was displaced prior to task failure being reached, preventing a valid twitch amplitude measurement. For these two trials, only those measures prior to the displacement were included in the analyses. In one instance, the last measured time point was ∼10 min into the trial, and in the other ∼15 min.

#### 2.4.3 Electromyography

Muscle activity of the quadriceps was measured using bipolar surface electromyography (EMG). Self‐adhesive Ag–AgCl surface electrode pairs (10 mm diameter; Kendall MediTrace), were placed over the muscle belly 20 mm apart (centre to centre) of the right vastus lateralis (VL) and rectus femoris (RF), just proximal to the myotendinous junction. Electrodes were placed in the assumed orientation of the fibres, with a ground electrode placed over the patella. Prior to placing the electrodes, the skin was shaved, abraded and cleansed with alcohol swabs to minimize resistance. EMG signals were recorded at a sampling rate of 2000 Hz using PowerLab (16/30‐ML880/P, ADInstruments), amplified, bandpass filtered (5–500 Hz), and analysed offline using LabChart 8 software (ADInstruments). The root mean square (RMS) of the VL and RF EMG signals was computed and the peak RMS was calculated for the 10 pedal revolutions prior to the three stimulations and averaged. Also, at each stimulation time point, the peak‐to‐peak amplitude of the three muscle compound action potentials (*M*_max_) for the VL and RF EMG were averaged to obtain one *M*_max_ for each muscle at each time point. The RMS *M*_max_^−1^ was then calculated for both VL and RF as the ratio of these values. Additionally, the mean power frequency (MPF) of the EMG signals was calculated across a 250 ms window surrounding the peak RMS value for the ten pedal revolutions prior to the stimulations and averaged. Finally, as both the RMS *M*_max_^−1^ and the MPF for VL and RF showed similar general trends across time, the values for these muscles were averaged at each time point to obtain a more global measure of quadriceps muscle activity.

#### 2.4.4 Muscle oxygenation

Muscle oxygenation ($S_{mO_{2}}$) of the right VL muscle was measured by a near‐infrared spectroscopy (NIRS) probe (MOXY Monitor, Fortiori Design LLC, Hutchinson, MN, USA), which was placed approximately halfway between the greater trochanter and the lateral epicondyle of the femur, and sampled at 2 Hz. The device was wrapped with an elastic bandage to minimize movement and limit external light interference. During the constant power output trials, 20 s bin averages for $S_{mO_{2}}$ were taken at identical time points as the ${\dot{V}}_{O_{2}}$ measurements (between 2:10 and 2:30 and 4:40 and 5:00 of each 5‐min period).

#### 2.4.5 Pedalling mechanical variables

Pedal force data were exported to LabChart (ADInstruments) for further analysis. The total force applied to the right pedal (*F*_total_) was calculated as the vector sum of the normal (*F*_normal_) and tangential (*F*_tangential_) force components. The effective force (*F*_effective_) is the component of the total force acting perpendicular to the crank (Dorel et al., 2009; Mornieux et al., 2006) (see Figure 1). It has been shown that the impact of pedalling technique on cycling efficiency may be made more apparent by differentiating the full pedal stroke into its downstroke (from 0° (top dead centre) to 180° (bottom dead centre)) and upstroke (from 180° to 360°) phases (Mornieux et al., 2006). Therefore, the primary pedal force variables of interest were the maximum *F*_total_ and *F*_effective_ during the downstroke (*F*_total‐max_ and *F*_effective‐max_, respectively) and the minimum *F*_total_ and *F*_effective_ during the upstroke (*F*_total‐min_ and *F*_effective‐min_, respectively). The index of effectiveness was calculated as the ratio of the mean *F*_effective_ to the mean *F*_total_ across a given crank interval, and this was calculated for the downstroke portion of the crank cycle (from 0° to 180°), the upstroke portion (from 180° to 360°), and the full revolution (from 0° to 360°) (Dorel et al., 2009; Mornieux et al., 2006). Finally, the evenness of force distribution was calculated as the ratio of the mean and maximum values of *F*_effective_ across the full pedal revolution (Korff et al., 2007). At each stimulation time point, all variables were measured across the 10 pedal revolutions prior to the stimulations (i.e., the same revolutions as the EMG variables) and averaged.

#### 2.4.6 Statistical analyses

Data are presented as mean (standard deviation). Statistical analyses were conducted using R v 4.3.0. To assess any general trends in variables across time, a linear mixed effects model was run using the *nlme* package in R using all data from both trials for each participant. For each variable of interest, time was included as a fixed effect, with the intercept and effect of time allowed to vary across participants. For each variable, if a significant effect of time was observed across the whole trial, a separate linear mixed model was fit to the common time points across all participants (up to 10 min and at task failure), and pairwise comparisons were made using the estimated marginal means with the *emmeans* package in R, with a Bonferroni–Holm correction applied. To explore the correlations between variables, a repeated measures correlation using the *rmcorr* package in R was performed. Repeated measures correlations were done both on the group level, to assess the common intra‐individual association between variables of interest, and on an individual level, to assess the common intra‐trial associations between variables for each participant. For two participants, NIRS signal quality prevented any analysis to be performed. Correlation coefficients were interpreted as negligible (0.00–0.09), weak (0.10–0.39), moderate (0.40–0.69), strong (0.70–0.89) and very strong (0.90–1.00) (Schober et al., 2018). Significance was set at α = 0.05.

## 3 RESULTS

**FIGURE 2:**
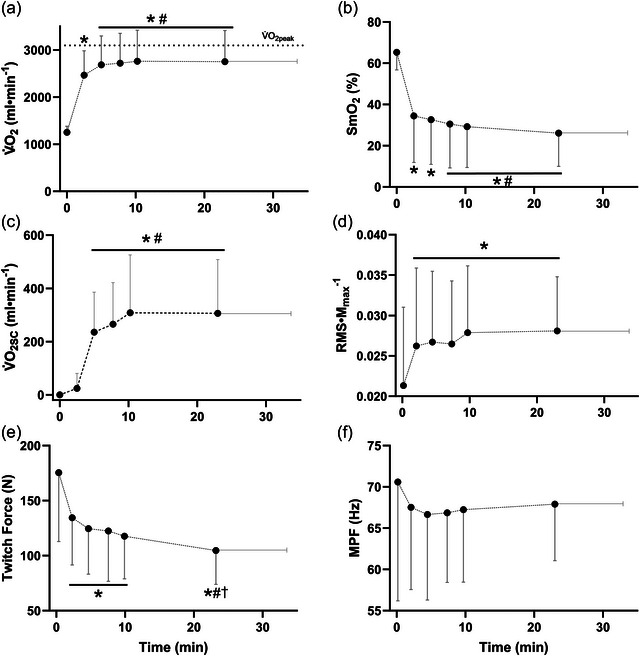
Physiological responses across a fatiguing cycling task. (a) Pulmonary oxygen uptake (${\dot{V}}_{O_{2}}$), (b) vastus lateralis muscle oxygenation ($S_{mO_{2}}$), (c) ${\dot{V}}_{O_{2}}$ slow component magnitude (${\dot{V}}_{O_{2}\mathrm{SC}}$), (d) EMG peak root mean square normalized to M‐wave, averaged between vastus lateralis and rectus femoris (RMS *M*_max_^−1^), (e) quadriceps twitch force, and (f) EMG mean power frequency (MPF), averaged between vastus lateralis and rectus femoris. Data are compiled across two sessions for *n* = 11 participants. Data shown are means ± SD up to the 10 min time point and at task failure. Across the entire trial duration, there were significant effects of time for all variables. *Significant difference from exercise onset. #Significant difference from 2.5 min. †Significant difference from 5 min.

${\dot{V}}_{O_{2}\mathrm{peak}}$ from the incremental test was 3.1 (0.7) L min^−1^. For the constant power output rides, peak ${\dot{V}}_{O_{2}}$ reached a mean of 3.0 (0.7) L min^−1^, or 98 (7)% of ${\dot{V}}_{O_{2}\mathrm{peak}}$ from the incremental test, which was not different from that measured during the incremental test (*P* = 0.364), and which occurred at an mean time of 15.8 (8.1) min. However, as a result of ${\dot{V}}_{O_{2}}$ decreasing immediately prior to task failure in several participants, the mean ${\dot{V}}_{O_{2}}$ at task failure was 2.8 (0.7) L min^−1^, which represented 90 (6)% of ${\dot{V}}_{O_{2}\mathrm{peak}}$ (Figure 2a). The mean duration of the trials was 23.6 (10.7) min (range of ∼10–55 min). As expected, during the constant power output trials, significant increases across time were found for ${\dot{V}}_{O_{2}}$ and ${\dot{V}}_{O_{2}\mathrm{SC}}$ (*P* ≤ 0.002). For both variables, pairwise comparisons showed significant increases between the 2.5 min and all later time points (*P* ≤ 0.001), but no changes were detected between 5 min and subsequent time points (*P* ≥ 0.131) (Figure 2a,c). $S_{mO_{2}}$ similarly decreased over time (*P* < 0.0001), with reductions occurring between 2.5 min and 7.5 min and 10 min and task failure (*P* ≤ 0.048), but once again there were no changes occurring beyond 5 min (*P* ≥ 0.061) (Figure 2b). Twitch force declined across time (*P *< 0.0001), with reductions occurring between exercise onset and 2.5 min (*P *= 0.012), and again between 2.5 and both 5 min and task failure (*P* ≤ 0.025). Unlike ${\dot{V}}_{O_{2}}$ and ${\dot{V}}_{O_{2}\mathrm{SC}}$, however, there were no changes in twitch force between 2.5 and 10 min (*P* ≥ 0.096) (Figure 2e). For the EMG variables, there was an increase over time in RMS *M*_max_^−1^ (*P* = 0.009), with changes occurring between exercise onset and all subsequent time points (*P *≤ 0.018), but no further increases were observed from 2.5 min onwards (*P *> 0.999) (Figure 2d). Finally, there was a significant reduction in MPF (*P* = 0.029), however pairwise comparisons failed to detect any changes across the first 10 min and task failure (*P *≥ 0.262) (Figure 2f). The mean (SD) values for these physiological variables for the common time points across all participants (up to 10 min and at task failure) are shown in Figure 2.

**FIGURE 3:**
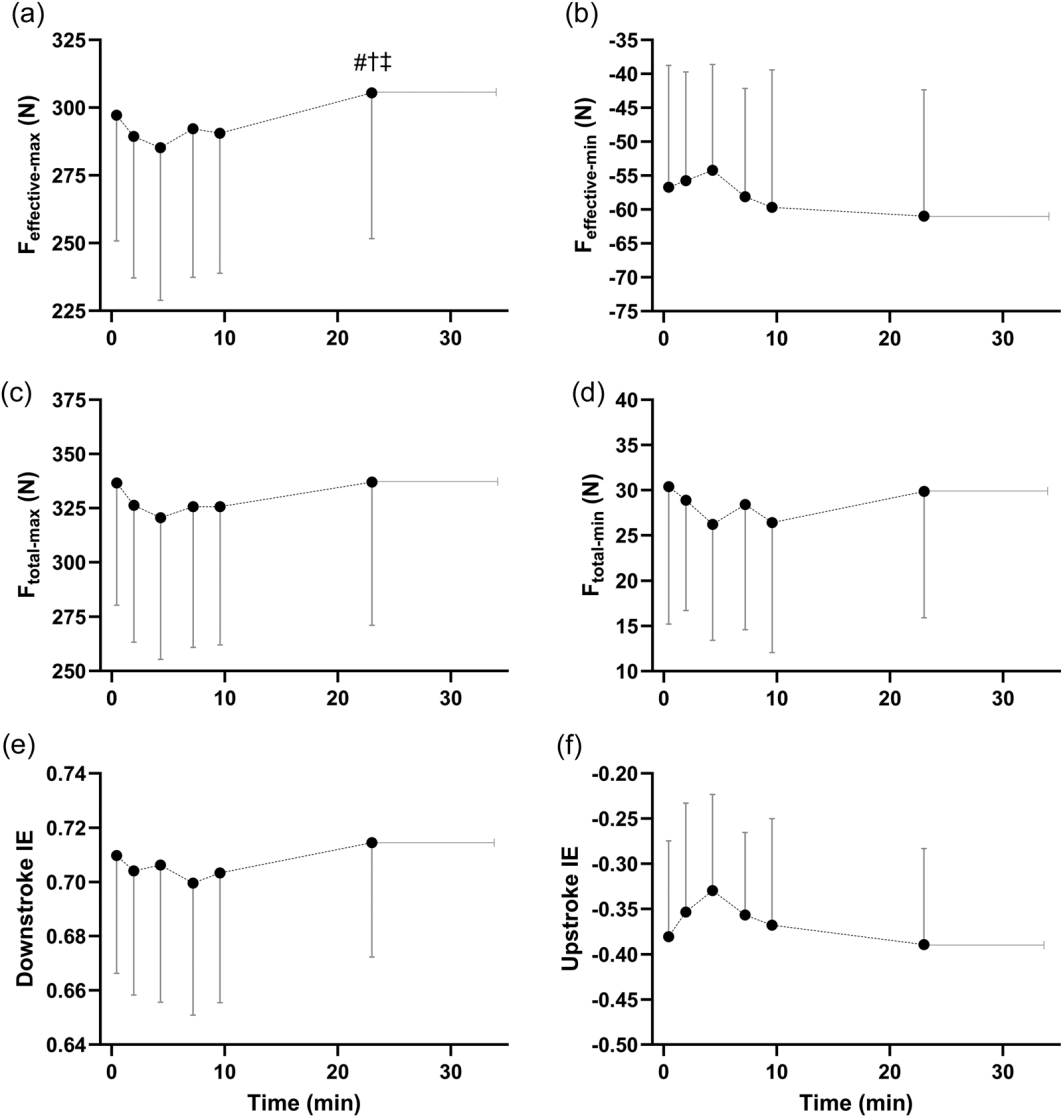
Mechanical variables for pedalling across a fatiguing cycling trial. (a) Maximum effective force on the downstroke (*F*_effective‐max_), (b) minimum effective force on the upstroke (*F*_effective‐min_), (c) maximum total force on the downstroke (*F*_total‐max_), (d) minimum total force on the upstroke (*F*_effective‐min_), (e) downstroke index of effectiveness (IE), and (f) upstroke index of effectiveness. Data are compiled across two sessions for *n* = 11 participants. Data shown are means ± SD up to the 10 min time point and at task failure. Across the entire trial duration, there were significant effects of time for *F*_effective‐max_, *F*_total‐max_, *F*_effective‐min_ and downstroke index of effectiveness. #Significant difference from 2.5 min. †Significant difference from 5 min. ‡Significant difference from 10 min.

For the pedal force variables, there was a significant increase across time for *F*_effective‐max_ (*P *= 0.005), where values at task failure were greater than at 2.5, 5 and 10 min (*P *≤ 0.029), with no other differences between time points observed (*P* ≥ 0.069) (Figure 3a). Significant time effects were also detected for *F*_total‐max_ (*P* = 0.043), *F*_effective‐min_ (*P* = 0.036) and the downstroke index of effectiveness (*P* = 0.026), although for these variables no significant pairwise comparisons emerged (*P* ≥ 0.123) (Figure 3b,c,e). No significant time effects were observed for *F*_total‐min_ (*P* = 0.229) (Figure 3d), upstroke index of effectiveness (*P* = 0.083) (Figure 3f), or evenness (*P* = 0.353). The mean (SD) values for these pedal force variables for the common time points up to 10 min and at task failure are shown in Figure 3.

**FIGURE 4:**
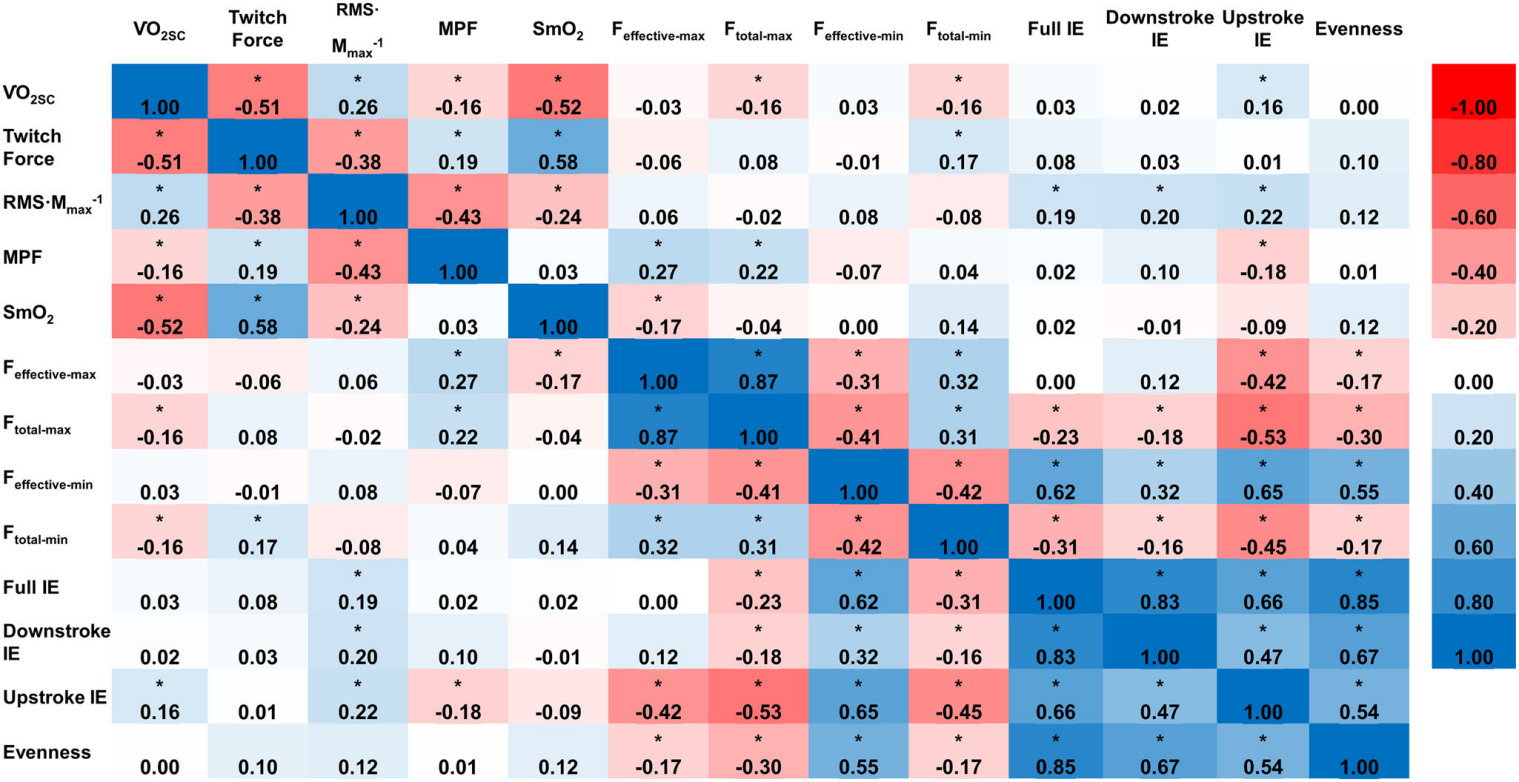
Repeated measures correlation matrix between physiological and mechanical variables across a fatiguing cycling trial. Data are compiled across two sessions for *n* = 11 participants. The reference scale showing colours associated with the respective strengths of correlation is shown on the far right side of the figure. *P < 0.05. *F*_effective‐min_, minimum effective pedal force exerted on the upstroke; *F*_total‐max_, maximum total pedal force exerted on the downstroke; *F*_total‐min_, minimum total pedal force exerted on the upstroke; IE, index of effectiveness; MPF, EMG mean power frequency, averaged between vastus lateralis and rectus femoris; RMS *M*_max_^−1^, EMG peak root mean square normalized to M‐wave, averaged between vastus lateralis and rectus femoris; $S_{mO_{2}}$, vastus lateralis muscle oxygenation; ${\dot{V}}_{O_{2}\mathrm{SC}}$, magnitude of ${\dot{V}}_{O_{2}}$ slow component; *F*_effective‐max_, maximum effective pedal force exerted on the downstroke.

**FIGURE 5:**
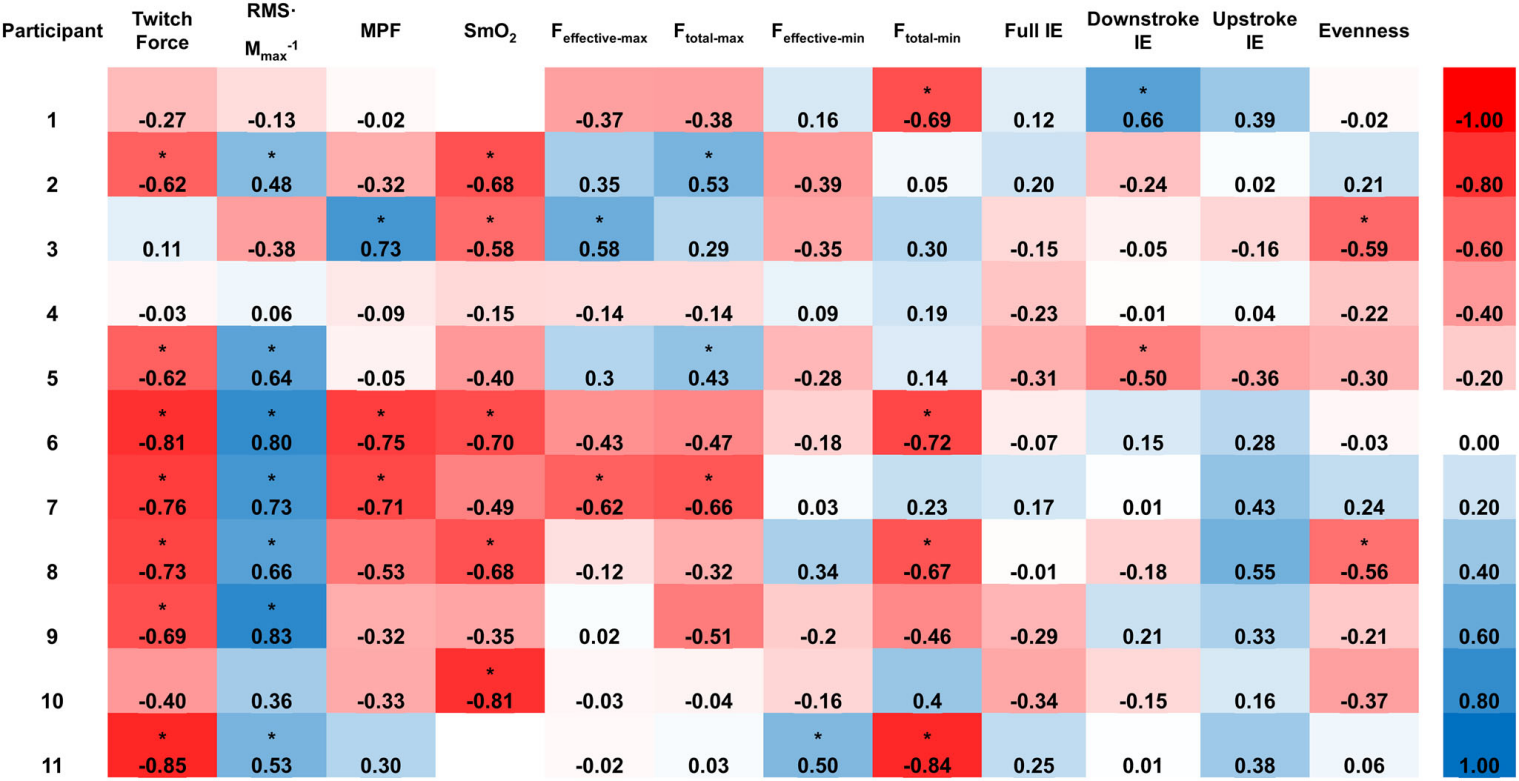
Individual correlations between the magnitude of the ${\dot{V}}_{O_{2}}$ slow component (${\dot{V}}_{O_{2}\mathrm{SC}}$) and other physiological and mechanical variables across a fatiguing cycling trial. For each participant, the repeated measures correlation was calculated indicating the common intra‐trial correlation between the magnitude of ${\dot{V}}_{O_{2}\mathrm{SC}}$ and the other listed variables. $S_{mO_{2}}$ correlations could not be made for participants one and six due to poor NIRS signal quality. For all other data, data are compiled across two sessions for *n* = 11 participants. The reference scale showing colours associated with the respective strengths of correlation is shown on the far right side of the figure. *P < 0.05. *F*_effective‐max_, maximum effective pedal force exerted on the downstroke; *F*_effective‐min_, minimum effective pedal force exerted on the upstroke; *F*_total‐max_, maximum total pedal force exerted on the downstroke; *F*_total‐min_, minimum total pedal force exerted on the upstroke; IE, index of effectiveness; MPF, EMG mean power frequency, averaged between vastus lateralis and rectus femoris; RMS *M*_max_^−1^, EMG peak root mean square normalized to M‐wave, averaged between vastus lateralis and rectus femoris; $S_{mO_{2}}$, vastus lateralis muscle oxygenation.

The group level repeated measures correlations between all variables are shown in Figure 4. Of the physiological variables, there were moderate negative correlations between ${\dot{V}}_{O_{2}\mathrm{SC}}$ and twitch force (*r*_rm_ = −0.51, *P *< 0.0001), and $S_{mO_{2}}$ (*r*_rm_ = −0.52, *P* < 0.0001), a weak negative correlation between ${\dot{V}}_{O_{2}\mathrm{SC}}$ and MPF (*r*_rm_ = −0.16, *P* = 0.023), and a weak positive correlation between ${\dot{V}}_{O_{2}\mathrm{SC}}$ and RMS *M*_max_^−1^ (*r*_rm_ = 0.26, *P* = 0.002). Of the pedalling mechanical variables, there were weak negative correlations between ${\dot{V}}_{O_{2}\mathrm{SC}}$ and *F*_total‐max_ (*r*_rm_ = −0.16, *P* = 0.020) and *F*_total‐min_ (*r*_rm_ = −0.16, *P* = 0.021), and a weak positive correlation between ${\dot{V}}_{O_{2}\mathrm{SC}}$ and the upstroke index of effectiveness (*r*_rm_ = 0.16, *P* = 0.020). Individual repeated measures correlations between ${\dot{V}}_{O_{2}\mathrm{SC}}$ and the other physiological and mechanical variables are shown in Figure 5.

## 4 DISCUSSION

This study is the first study to assess muscle fatigue in real‐time during a cycling task to the limit of endurance. While improving temporal resolution of the assessment of fatigue, we have also avoided potential confounding factors such as the recovery of muscle force that may occur when using traditional isometric dynamometers for post‐exercise fatigue assessments. Here, we report weak to moderate correlations between the magnitude of ${\dot{V}}_{O_{2}\mathrm{SC}}$ and muscle fatigue, muscle oxygenation and quadriceps EMG activity, when calculated over the entire group. However, the time course of fatigue was quite different from the time course of the slow component of ${\dot{V}}_{O_{2}}$. Secondly, there were only weak correlations between the slow component of ${\dot{V}}_{O_{2}}$ and alterations in several aspects of pedalling technique, such as the force exerted during the upstroke portion of the pedal stroke. However, there were some strikingly high correlations at the individual level for some of these mechanical measures. These data highlight the complexities of ${\dot{V}}_{O_{2}\mathrm{SC}}$, and suggest that it may be a multifaceted phenomenon having both physiological and biomechanical mechanisms.

### 4.1 Physiological correlates of the ${\dot{V}}_{O_{2}}$ slow component

Despite having been extensively studied for over 50 years, the exact physiological mechanism of ${\dot{V}}_{O_{2}\mathrm{SC}}$ is still a matter of debate in the scientific community. It is generally believed that the majority (∼85%) of the increase in ${\dot{V}}_{O_{2}}$ during fatiguing exercise is a result of increases in oxygen consumption within the working musculature, rather than increases in ventilatory, cardiac or auxiliary muscle activity (Jones et al., 2011; Poole et al., 1991). While we did not measure muscle ${\dot{V}}_{O_{2}}$ directly, the NIRS data allow us an approximation of changes in muscle ${\dot{V}}_{O_{2}}$ during exercise, and indeed, the continual decrease in $S_{mO_{2}}$ evident in Figure 2 suggests that the increase in pulmonary ${\dot{V}}_{O_{2}}$ associated with the slow component coincides with increased oxygen consumption within the working muscles. The correlations reported here are similar to those between the magnitude of the slow component and the NIRS deoxyhemoglobin signal of the VL reported by Özyener et al. (2012).

Acknowledging the source of the increase in ${\dot{V}}_{O_{2}}$ as being predominantly from the working musculature, it has been a natural assumption that the development of ${\dot{V}}_{O_{2}\mathrm{SC}}$ may be mechanistically linked to the underlying fatigue processes where, for example, the apparent increase in oxygen cost may be due to the recruitment of presumably less efficient fast twitch fibres as fatigue progresses (Whipp, 1994). Here, we observed a general increase in RMS *M*_max_^−1^, consistent with the recruitment of additional motor units, as well as a reduction in MPF, reflecting reductions in muscle fibre conduction velocity that accompany fatigue. As a more direct measure of muscle fatigue, we observed a significant reduction in twitch force while cycling. As seen in Figure 2, fatigue was strongly evident in the first 2.5 min, while the onset of the slow component was often only apparent after this time. Our results align well with the general findings of both Cannon et al. (2011) and do Nascimento Salvador et al. (2023); the latter authors actually reported no significant relationship between muscle force decline and the slow component. These authors both reported significant reductions in cycling‐specific peak power output in the first 2−3 min of exercise, with no further reductions in the subsequent 8−12 min, despite ${\dot{V}}_{O_{2}}$ continuing to rise over this period. Similarly, in the present study, there were no significant changes in either twitch force or RMS *M*_max_^−1^ between 2.5 and 10 min, despite a significant increase in ${\dot{V}}_{O_{2}}$ across this time. It seems that the greatest alterations in muscle fatigue generally occur in the early periods of high‐intensity exercise, prior to the onset of the slow component, but do not seem to closely follow the rise in ${\dot{V}}_{O_{2}}$ observed thereafter. The strength of the association between twitch force and the slow component reported here (*r*_rm_ = −0.51) is within the (notably wide) range of correlations others have reported, which, depending on the specific measure of fatigue used, range from *r^2^* values of ∼0.20−0.35 on the low end (Cannon et al., 2011; de Almeida Azevedo et al., 2022; do Nascimento Salvador et al., 2018; Keir et al., 2016) to ∼0.65–0.80 on the high end (de Almeida Azevedo et al., 2022; Gajanand et al., 2020; Keir et al., 2016). These disparities, along with the heterogeneity in the strength of the association within the individual analyses reported here, would suggest that fatigue may only play a relatively small role in its development overall, and/or that the relationship between fatigue and the slow component is not a causal one. Here it is also worth noting that Hopker et al. (2016) found that fatigue induced by a non‐metabolically stressful protocol of 100 drop jumps had no impact on ${\dot{V}}_{O_{2}}$ kinetics or the magnitude of the slow component, suggesting that simply muscular fatigue is not enough to explain the rise in ${\dot{V}}_{O_{2}}$ associated with the slow component.

### 4.2 Mechanical correlates of the ${\dot{V}}_{O_{2}}$ slow component

One of our primary interests in this study was to explore if alterations in pedalling technique during a fatiguing cycling trial could explain any of the observed increases in ${\dot{V}}_{O_{2}}$ associated with the slow component. In this realm, both Dorel et al. (2009) and Sanderson and Black (2003) reported significant increases in the maximum effective force exerted on the pedals of between ∼6% and 11%, respectively, between the beginning and end of a fatiguing cycling trial lasting approximately 13 min. It seems reasonable that this increased force production at a given power output could conceivably necessitate a greater energy demand, contributing to the rise in ${\dot{V}}_{O_{2}}$ observed with the slow component. Our analysis showed a significant increase over time in the maximum effective force; however, similar to Dorel et al. (2009), any consistent increases in this variable did not occur until later in the exercise bout, and here it was not correlated with ${\dot{V}}_{O_{2}\mathrm{SC}}$.

Instead, the repeated measures correlation analysis seemed to provide a different perspective on the changing mechanical variables. At a group level, we observed a significant but weak negative correlation between ${\dot{V}}_{O_{2}\mathrm{SC}}$ and both the maximum total force exerted on the downstroke and the minimum total force exerted on the upstroke. Additionally, we found a significant positive correlation between ${\dot{V}}_{O_{2}\mathrm{SC}}$ and the upstroke index of effectiveness. While at a group level, we could not detect any systematic changes in these variables across time, Figure 3 illustrates that the maximum or minimum values for these variables occurred around the 5 min time point, the same time point as that of the largest relative increase in the slow component. Any decrease in minimum effective force on the upstroke would be consistent with participants creating a relatively greater upward force during the upstroke portion of the pedal cycle, which, notably, aligns well with prior studies showing the impact of modifying cycling technique on efficiency. For example, Korff et al. (2007) found that when participants were instructed to actively pull up during the upstroke portion of the crank cycle, gross efficiency was significantly reduced compared to when participants cycled with their preferred technique. Additionally, Cannon et al. (2007) reported a reduction in gross efficiency when participants were instructed to maintain a dorsiflexed ankle position throughout the crank cycle, which may be speculated to have a similar mechanical impact.

**FIGURE 6:**
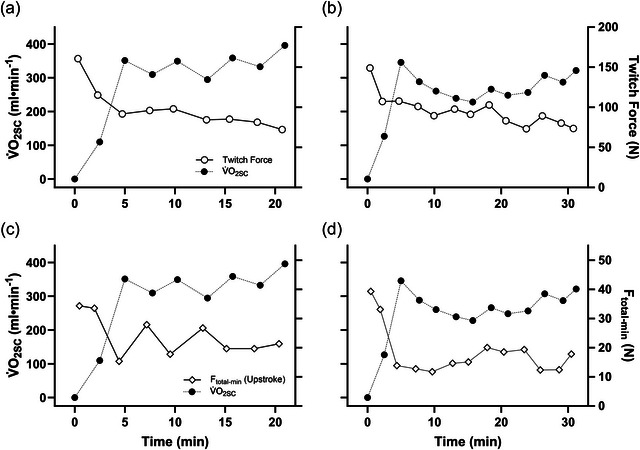
The interplay between alterations in fatigue, pedalling technique and the slow component during cycling. To illustrate the temporal association between variables, the magnitude of the ${\dot{V}}_{O_{2}}$ slow component (${\dot{V}}_{O_{2}\mathrm{SC}}$) and twitch force are overlaid in (a, b), while that of the ${\dot{V}}_{O_{2}\mathrm{SC}}$ and the minimum total pedal force exerted on the upstroke (*F*_total‐min_) are shown in (c, d). (a) and (c) correspond to the first trial, and (b) and (d) correspond to the second trial. For this participant, both twitch force (*r*_rm_ = −0.85) and *F*_total‐min_ (*r*_rm_ = −0.84) were similarly correlated with ${\dot{V}}_{O_{2}\mathrm{SC}}$.

Figure 6 shows the interplay between alterations in ${\dot{V}}_{O_{2}\mathrm{SC}}$, muscle fatigue and pedalling technique in participant 11, for whom there were high correlations between the slow component and both twitch force and *F*_total‐min_. It is interesting to note the apparent congruency between alterations in *F*_total‐min_ and the concomitant subtle rising and falling in ${\dot{V}}_{O_{2}}$ across the two trials. While the individual heterogeneity in the correlations we observed would question the importance of these changes in explaining a large fraction of the slow component observed during cycling overall, this example would suggest that for some individuals, changes in pedalling mechanics may act as a factor affecting energy cost, which may then be superimposed on the slow component of ${\dot{V}}_{O_{2}}$. While it is difficult to estimate the relative energetic ‘impact’ of these changes, as one point of comparison, Cannon et al. (2007) reported a significant increase in ${\dot{V}}_{O_{2}}$ of ∼75 mL min^−1^ when participants were instructed to maintain a dorsiflexed ankle position compared to their preferred technique, while Korff et al. (2007) reported increases in ${\dot{V}}_{O_{2}}$ of ∼200 mL min^−1^ when participants were instructed to actively pull up on the upstroke. Considering the typical range of slow component magnitudes, these values would seem to be in the realm of possibility.

### 4.3 Limitations

While the novel fatigue assessment utilized in the current study provides advantages over other common methods due to its non‐disruptive nature and allowance for a higher temporal resolution of muscle fatigue, it is not without its limitations. First of all, it may be noted that the data used in the current study were obtained from an adjacent study establishing the validity and reliability of the fatigue assessment method (MacDougall et al., 2024), which required brief (15–30 s) resting periods every ∼5 min through the task, in which isometric twitch measurements were obtained. Although following these breaks subsequent fatigue measurements were not performed until several minutes into exercise, considering that muscle fatigue recovery may be rapid, we cannot rule out that these breaks altered the trajectory of muscle fatigue across the trials. Second, it is important to acknowledge that exercise‐induced fatigue is known to be task‐dependent (Enoka & Duchateau, 2016) and so it may be argued that, for example, measuring reductions in cycling‐specific power output may be the most appropriate measure of cycling‐induced fatigue (e.g., Cannon et al., 2011; do Nascimento Salvador et al., 2018, 2023). However, performing maximal sprints (or any other maximal physical effort) across a cycling task is not only disruptive to the task itself, limiting the temporal resolution of the measure, but also may itself be fatiguing, and so could also confound the development of fatigue from the task itself if done multiple times through a task. Finally, while the measurement of electrically evoked quadriceps force is a common method of assessing muscle fatigue resulting from cycling exercise (de Almeida Azevedo et al., 2022; Gajanand et al., 2020; Keir et al., 2016), it obviously can only capture the fatigue occurring in a single muscle group. Considering that several other muscle groups, such as the hip extensors and ankle plantar flexors, may also play a primary role in pedalling power production (and thus be subject to fatigue as well) (Ericson et al., 1986), the assessment of solely quadriceps fatigue may limit the extent to which we can make strong conclusions on the relationship between muscle fatigue and ${\dot{V}}_{O_{2}\mathrm{SC}}$ during cycling. Nonetheless, it may again be highlighted that our results agree well with prior studies which did include cycling‐specific measures of fatigue (Cannon et al., 2011; do Nascimento Salvador et al., 2023), suggesting that our measure of fatigue was able to capture similar information to that done in a more task‐specific manner.

### 4.4 Conclusion

Overall, it is difficult to reconcile the many discrepant findings in the literature regarding the mechanisms of ${\dot{V}}_{O_{2}\mathrm{SC}}$. For every study lending support to a particular hypothesis, there seems to be at least one other which directly refutes it. In the present study, we add to existing literature showing that the time course of fatigue does not match the evolution of the slow component of ${\dot{V}}_{O_{2}}$, and which is underscored by a wide range in the strength of associations at an individual level. Our observations on the relationship between ${\dot{V}}_{O_{2}\mathrm{SC}}$ and pedalling technique revealed only a weak correlation in general to alterations in cycling mechanics consistent with relying on a relatively greater upward pulling action through the upstroke, but provide isolated examples where changes in mechanics may contribute to changes in energy cost. Evidently, more research is needed to disentangle the complex interplay between potentially many factors, both physiological and biomechanical, that may be related to the development of ${\dot{V}}_{O_{2}\mathrm{SC}}$.

## AUTHOR CONTRIBUTIONS

Keenan B. MacDougall, Saied J. Aboodarda, and Brian R. MacIntosh conceived the idea for the project and contributed to the design of the protocol. Keenan B. MacDougall and Paulina H. Westergard collected and analysed the data. Keenan B. MacDougall drafted the manuscript, and Saied J. Aboodarda, Paulina H. Westergard, and Brian R. MacIntosh revised it critically. All authors approved the final version of the manuscript and agree to be accountable for all aspects of the work in ensuring that questions related to the accuracy or integrity of any part of the work are appropriately investigated and resolved. All persons designated as authors qualify for authorship, and all those who qualify for authorship are listed.

## CONFLICT OF INTEREST

None declared.

## Data Availability

Data generated or analysed during this study are available from the corresponding author upon reasonable request.
